# Supplementary material for: Plasma Oxidative Status in Preterm Infants Receiving LCPUFA Supplementation: A Pilot Study
Source: Nutrients. 2020 Jan 1;12(1):122. doi: 10.3390/nu12010122 (PMC7019959; doi:10.3390/nu12010122)
Supplement: Supplementary file 1 [file nutrients-12-00122-s001.pdf]

Table S1. Long-chain polyunsaturated fatty acids at birth according to infant sex.

	**Male (*n* = 8)**	**Female (*n* = 13)**	***p*-value**
**Long-chain polyunsaturated fatty acids**			
Linoleic acid *(nmol/ml)*	1395.6 (1617)	850.8 (566)	*0.624*
Dihomo-γ-linolenic acid *(nmol/ml)*	137.9 (71.5)	104.9 (38.6)	*0.135*
Arachidonic acid *(nmol/ml)*	659.2 (324.8)	516.4 (239.4)	*0.181*
α-linolenic acid *(nmol/ml)*	69.0 (141.3)	24.4 (42.9)	*0.970*
Eicosapentaenoic acid *(nmol/ml)*	155.1 (260.0)	69.2 (70.0)	*0.851*
Docosahexaenoic acid *(nmol/ml)*	268.1 (306.3)	221.9 (91.5)	*0.624*
**Antioxidants**			
Catalase activity *(U cata/mg protein)*	4.4 (1.8)	3.4 (3.0)	0.860
Thiol groups *(mM GSH/mg protein)*	0.01 (0.0)	0.01 (0.0)	0.697
Reduced glutathione *(mg GSH/mg protein)*	0.2 (0.2)	0.2 (0.1)	0.595
Antiox-S *(arbitrary units)*	0.1 (0.6)	-0.1 (1.5)	0.595
**Biomarkers of oxidative damage**			
Malondialdehyde *(μmol/ml)*	6.0 (2.8)	4.6 (2.2)	0.145
Total carbonyl proteins *(nmol/mg protein)*	0.4 (0.3)	0.3 (0.3)	0.268
Oxy-S *(arbitrary units)*	−0.6 (0.7)	0.03 (1.0)	0.213

Data show medians (IQR). Mann–Whitney U test.

Table S2. Increases in anthropometric parameters and growth velocities from birth to end of supplementation.

	**MCT-S (*n* = 12)**	**ARA:DHA-S (*n* = 10)**	***p*-value**
Δ Weight *(g)*	(509.8)	965.0 (491.5)	*0.445*
Δ Weight *z*-score	−1.53 (0.36)	−1.40 (0.44)	*0.428*
Δ Length *(cm)*	(1.7)	6.5 (4.2)	*0.547*
Δ Length *z*-score	−1.43 (4.27)	−1.37 (1.08)	*0.420*
Δ Head circumference *(cm)*	6.1 (2.2)	6.0 (3.7)	*0.690*
Δ Head circumference *z*-score	−0.72 (1.01)	−1.23 (0.63)	*0.421*
Weight gain *(g/kg/day)*	4.47)	10.43 (4.19)	*0.792*
Length gain *(cm/day)*	0.04)	0.14 (0.10)	*0.222*
Head circumference gain *(cm/day)*	0.09 (0.05)	0.11 (0.04)	*0.548*

Data show medians (IQR). Change (Δ). Mann–Whitney U test.
